# Predicting the Risk of Type 2 Diabetes Mellitus with the New Chinese Diabetes Risk Score in a Cohort Study

**DOI:** 10.3389/ijph.2023.1605611

**Published:** 2023-04-25

**Authors:** Hongen Chen, Yuhang She, Shuhong Dai, Li Wang, Na Tao, Shaofen Huang, Shan Xu, Yanmei Lou, Fulan Hu, Liping Li, Changyi Wang

**Affiliations:** ^1^ Department of Non-Communicable Disease Prevention and Control, Shenzhen Nanshan Center for Chronic Disease Control, Shenzhen, China; ^2^ Injury Prevention Research Center, Shantou University Medical College, Shantou, China; ^3^ School of Public Health, Shantou University, Shantou, China; ^4^ Department of Pharmacy, Affiliated Hospital of Zunyi Medical University, Zunyi, Guizhou, China; ^5^ Shenzhen Nanshan District Shekou People’s Hospital, Shenzhen, China; ^6^ Department of Health Management, Beijing Xiao Tang Shan Hospital, Beijing, China; ^7^ Department of Epidemiology and Health Statistics, School of Public Health, Shenzhen University Health Science Center, Shenzhen, China

**Keywords:** screening, cohort study, China, T2DM, new Chinese diabetes risk score

## Abstract

**Objectives:** The New Chinese Diabetes Risk Score (NCDRS) is a noninvasive tool to assess the risk of type 2 diabetes mellitus (T2DM) in the Chinese population. Our study aimed to evaluate the performance of the NCDRS in predicting T2DM risk with a large cohort.

**Methods:** The NCDRS was calculated, and participants were categorized into groups by optimal cutoff or quartiles. Hazard ratios (HRs) and 95% confidential intervals (CIs) in Cox proportional hazards models were used to estimate the association between the baseline NCDRS and the risk of T2DM. The performance of the NCDRS was assessed by the area under the curve (AUC).

**Results:** The T2DM risk was significantly increased in participants with NCDRS ≥25 (HR = 2.12, 95% CI 1.88–2.39) compared with NCDRS <25 after adjusting for potential confounders. T2DM risk also showed a significant increasing trend from the lowest to the highest quartile of NCDRS. The AUC was 0.777 (95% CI 0.640–0.786) with a cutoff of 25.50.

**Conclusion:** The NCDRS had a significant positive association with T2DM risk, and the NCDRS is valid for T2DM screening in China.

## Introduction

Diabetes is one of the most challenging public health problems worldwide (1). The International Diabetes Federation (IDF) reported that approximately 1 in 11 adults aged 20–79 years were living with diabetes globally, and 1 in 2 adults with diabetes were undiagnosed (2). The prevalence of diabetes in Chinese adults has increased to 11.2%, and the awareness rate, treatment rate, and control rate of diabetes are quite low, making diabetes one of the most serious chronic diseases in this country (3). Type 2 diabetes mellitus (T2DM), as the main type of diabetes patients, is preventable through multiple intervention programs (4–7). Therefore, an early approach to detect T2DM by simple screening tools, to provide timely interventions to individuals at high risk of developing T2DM, could reduce the risk of complications and be more cost-effective than current T2DM management approaches (4, 8).

A series of well-designed risk score models have been developed in recent years for screening the population for undiagnosed T2DM. Generally, these models are composed of a few variables that can easily be obtained through medical health checks, and can predict the T2DM risk by adding up the scores of each variable (9). The Finnish Diabetes Risk score (FINDRISC) (10, 11), Cambridge Risk Score (CRS) (12), QDiabetes (13), and the Australian Type 2 Diabetes Risk Assessment Tool (AUSDRISK) are popular models used for T2DM risk prediction (14). However, previous studies were either limited by an inadequate sample size (10, 14), ethnicity derived from the Caucasian population (10–13), or validation only in a cross-sectional study (10, 11, 14). Further, previous Chinese risk score models for screening T2DM either failed to report important indicators (e.g., sex, family history of diabetes), consisted of clinical measurements, or even had low predictive values (15–17). A new simple and accurate T2DM risk prediction tool for the Chinese population is urgently needed to identify individuals with T2DM or at risk of T2DM, given the great burden that T2DM causes, enabling implementation of large scale T2DM screening in China (18).

The New Chinese Diabetes Risk Score (NCDRS) is a non-invasive T2DM assessment tool that includes 6 risk predictors: age, BMI, sex, waist circumference, systolic blood pressure, and family history of diabetes (19). The NCDRS was reported to be a reliable tool for detecting undiagnosed T2DM in a previous cross-sectional study conducted in Eastern China (20). However, to our knowledge, all of the studies that evaluated the NCDRS were cross-sectional (20–23), and cohort studies investigating the performance of NCDRS are missing. Whether the NCDRS performs well in recognizing undiagnosed T2DM needs to be validated in a large cohort study. Therefore, we aimed to evaluate the association between the NCDRS and the risk of T2DM to determine its performance in predicting T2DM risk based on a large prospective cohort study.

## Methods

### Study Design and Population

This was a cohort study conducted in Xiaotangshan Hospital, Beijing, China from 1 January 2009 to 31 December 2016. A total of 41,449 participants who underwent a comprehensive annual or biennial health examination were diagnosed without T2DM, and completed at least one follow-up were eligible for enrollment in this study. Among them, we further excluded participants if any of the following criteria were met: 1) a history of myocardial infarction, stroke, coronary heart disease, heart failure (*n* = 1,377), or cancer (*n* = 507); and 2) aged <20 and ≥80 years (*n* = 804). Ultimately, we included 39,538 adults in this study with a median follow-up of 37.47 months. Variables with >5% missing data were imputed using the method of multiple imputation (24).

The study was conducted in accordance with the principles of the Declaration of Helsinki (revised in 2013) (25) and it was approved by the Institutional Review Board of Xiaotangshan Hospital (No. 202006). Written informed consent was obtained from each participant.

### Data Collection

We used a standardized, self-administered questionnaire to obtain the demographic characteristics including age, sex, smoking status, alcohol consumption, history of diabetes, family history of diabetes, history of hypertension, history of cardiovascular disease, history of cancer, history of fatty liver disease (FLD), history of taking blood-lipid lowering drugs, and history of taking coronary heart disease (CHD) drugs. Smokers were defined as those who had smoked ≥100 cigarettes in life, alcohol consumption was defined as drinking ≥12 times in the past year (26), and family history of diabetes was defined as having at least one first-degree relative with diabetes. Each participant answered the questionnaire by themselves or with assistance in a face to face interview if they had difficulty completing the survey.

Height and weight were measured with a standard method while wearing light clothing and no shoes. Waist and hip circumference (WC and HC) were measured using a non-stretchable tape meter by trained nurses. WC and HC were measured as the narrowest level between the lower rib and the iliac crest and at the level of the greatest protrusion of the buttocks, respectively, with participants standing erect with their feet together. The measurement of blood pressure was performed three times repeatedly at the heart level on the right arm using an electronic sphygmomanometer (HEM-770AFuzzy, Omron, Japan) after being seated for at least 5 minutes of rest with 1-minute intervals between measurements. The final systolic blood pressure (SBP) and diastolic blood pressure (DBP) were defined as the mean of the 3 measurements. Body mass index (BMI) was calculated as the ratio of weight to height squared (kg/m^2^).

Blood samples were collected at 7–10 AM after an overnight fasting (at least 8 h) for the laboratory biochemical tests. The clinical indicators were analyzed including: serum uric acid (UA), creatinine (Cr), total cholesterol (TC), triglycerides (TG), low-density lipoprotein cholesterol (LDL-C), high-density lipoprotein cholesterol (HDL-C), alanine aminotransferase (ALT), aspartate aminotransferase (AST), fasting plasma glucose (FPG), white blood cell count (WBC), eosinophil count (EOS), eosinophil percentage (EOSP), erythrocyte sedimentation rate (ESR), mean corpuscular volume (MCV), and estimated glomerular filtration rate (eGFR). All of these indicators were performed in the same laboratory using standard laboratory methods, and certain established methods were performed as follows:

(1) The levels of UA, Cr, ALT, AST, TC, TG, LDL-C, and HDL-C were measured using enzymatic colorimetric methods in an automatic analyzer (Type 7600; Hitachi, Tokyo).
(2) FPG was tested *via* the glucose dehydrogenase method (Merck, Darmstadt, Germany).
(3) eGFR was evaluated as:
  eGFR = 175 × Cr−1.234 × age−0.179 [if woman, × 0.79]

### New Chinese Diabetes Risk Score (NCDRS)

The evaluation of the NCDRS has previously been reported (19). Briefly, the total score was obtained by adding up the six indicators, including age, BMI, sex, WC, SBP, and family history of diabetes, which ranged from 0 to 51. The optimal cutoff point to screen for type 2 diabetes was found to be 25. Participants were also categorized into 4 groups according to the NCDRS quartiles: <15, 15∼22, 22∼29, and ≥29.

### Diagnosis of T2DM

T2DM (ADA 2015 criteria) was defined by any of the following criteria being met: 1) history of T2DM; 2) current use of antidiabetic drugs; 3) FPG ≥7.0 mmol/L; 4) 2 h plasma glucose≥11.1 mmol/L; and 5) HbA1c ≥6.5%.

### Statistical Analysis

Baseline demographic characteristics of all participants were categorized by the optimal cutoff of the NCDRS. Continuous variables were described as the mean ± standard deviation (SD) or median ± interquartile range (IQR) depending on whether they were normally distributed. Frequency (%) was expressed for categorical variables. We applied Student’s t tests, Mann‒Whitney U tests, and Chi-square tests for group comparisons of normally distributed, skew-distributed, and categorical data, respectively. The person-years were calculated from the first cohort entry date to either the date of diagnosis of T2DM or the end of follow-up. We estimated the association between the baseline NCDRS and the risk of T2DM, with corresponding hazard ratios (HRs) and corresponding 95% confidential intervals (CIs) in univariable and multivariable Cox proportional hazards models. The lowest quartile was considered as a reference.

To reduce the potential confounders at baseline, three models with increasing degrees of adjustment were established. Model 1 was unadjusted. Model 2 was adjusted for covariates including Glu, smoking status, and alcohol consumption, and Model 3 was additionally adjusted for TG, TC, HDL, ALT, AST, eGFR, UA, heart rate, and white blood cell count (WBC). *P* for trend was used to evaluate the linear trend among the NCDRS quartiles as a continuous variable and the risk of T2DM in Cox models. We used the restricted cubic spline (RCS) to assess the dose‒response relationship between NCDRS and the incidence of T2DM. The area under the curve (AUC) was used to assess the performance of the NCDRS in predicting T2DM.

We examined the robustness of the results by performing sensitivity analyses that excluded participants who were diagnosed with T2DM within the first 2 years of follow-up. Subgroup analyses were also performed by stratifying participants by sex, hypertension, and fatty liver disease (FLD) to determine whether the association between the NCDRS and the risk of T2DM was stable in different groups.

All statistical analyses were performed with R 3.5.2 (R Foundation), with a two-sided *p* < 0.05 considered statistically significant.

## Results

### Baseline Characteristics of the Participants

The baseline characteristics of the participants categorized by the optimal cutoff of the NCDRS are presented in Table 1. BMI, heart rate, waist circumference, hip circumference, UA, Cr, TC, TG, LDL-C, ALT, AST, Glu, SBP, DBP, WBC, EOS, EOSP, ESR, MCV, and eGFR were statistically higher in the participants with NCDRS ≥25 (all *p* < 0.05), while HDL-C was statistically lower in the participants whose NCDRS ≥25 compared to the group of NCDRS <25 (*p* < 0.001). Among the participants, those with NCDRS ≥25 were more likely to be men or alcohol users. Hypertension, fatty liver disease, the use of blood-lipid lowering drugs or CHD drugs were more common in the NCDRS ≥25 group (all *p* < 0.001).

**TABLE 1:** Baseline characteristics of study participants across the New Chinese Diabetes Risk Score (Beijing, China. 2009–2016) (*n* = 39,538).

Baseline characteristics	NCDRS		*p*-value
	<25	≥25	
No. of participants	23,068	16,470	
BMI	23.23 (3.04)	27.01 (2.99)	<0.001
Heart rate, mean (SD), beats/min	75.52 (9.82)	76.42 (10.00)	<0.001
Waist circumference, mean (SD), cm	77.82 (8.79)	90.77 (7.89)	<0.001
Hip circumference, mean (SD), cm	94.01 (5.57)	99.27 (5.67)	<0.001
UA, mean (SD), μmol/L	304.39 (82.76)	363.13 (83.57)	<0.001
Cr, median (IQR), μmol/L	74.1 (64.60–86.40)	85.05 (74.60–94.00)	<0.001
TC, mean (SD), mmol/L	4.62 (0.86)	5.1 (0.93)	<0.001
TG, median (IQR), mmol/L	0.98 (0.71–1.45)	1.58 (1.12–2.28)	<0.001
LDL-C, mean (SD), mmol/L	2.77 (0.71)	3.17 (0.75)	<0.001
HDL-C, mean (SD), mmol/L	1.43 (0.34)	1.29 (0.29)	<0.001
ALT, median (IQR), U/L	16.00 (12.00–23.00)	22.60 (16.90–32.00)	<0.001
AST, median (IQR), U/L	18.00 (15.80–21.90)	21.00 (18.00–25.10)	<0.001
Glu, mean (SD), mmol/L	5.05 (0.43)	5.44 (0.52)	<0.001
SBP, mean (SD), mm Hg	111.04 (12.02)	127.88 (15.29)	<0.001
DBP, mean (SD), mm Hg	69.72 (8.40)	80.6 (9.80)	<0.001
WBC, median (IQR)	5.72 (4.89–6.77)	6.07 (5.20–7.14)	<0.001
EOS, median (IQR)	0.12 (0.08–0.18)	0.15 (0.10–0.23)	<0.001
EOSP, median (IQR), (%)	2.10 (1.40–3.10)	2.50 (1.70–3.70)	<0.001
ESR, mean (SD)	7.04 (6.56)	7.21 (6.91)	0.01
MCV, mean (SD)	90.36 (5.56)	91.31 (5.8)	<0.001
eGFR, mean (SD), mL/min/1.73 m^2^	66.83 (22.65)	72.52 (21.24)	<0.001
Gender, No. (%)			<0.001
Male	9,664 (0.44)	12,538 (0.56)	
Female	13,404 (0.77)	3,932 (0.23)	
Hypertention, No. (%)			<0.001
No	22,041 (0.71)	9,178 (0.29)	
Yes	1,027 (0.12)	7,292 (0.88)	
Fatty liver disease, No. (%)			<0.001
No	19,049 (0.72)	7,372 (0.28)	
Yes	4,019 (0.31)	9,098 (0.69)	
Blood-lipid lowering drugs, No. (%)			<0.001
No	23,046 (0.59)	16,343 (0.41)	
Yes	22 (0.15)	127 (0.85)	
CHD drugs, No. (%)			<0.001
No	23,027 (0.59)	15,991 (0.41)	
Yes	41 (0.08)	479 (0.92)	
Smoking status, No. (%)			<0.001
No	12,317 (0.63)	7,379 (0.37)	
Yes	10,751 (0.54)	9,091 (0.46)	
Alcohol consumption, No. (%)			<0.001
No	14,969 (0.67)	7,355 (0.33)	
Yes	8,099 (0.47)	9,115 (0.53)	

Note: Data are mean (SD), median (IQR) and percentage. Abbreviations: UA, uric acid; Cr, creatinine; TC, total cholesterol; TG, triglycerides; LDL-c, low-density lipoprotein cholesterol; HDL-c, high-density lipoprotein cholesterol; ALT, alanine aminotransferase; AST, aspartate aminotransferase; FPG, fasting plasma glucose; SBP, systolic blood pressure; DBP, diastolic blood pressure; WBC, white blood cell count; EOS, eosinophil; EOSP, eosinophil percentage; ESR, erythrocyte sedimentation rate; MCV, mean corpuscular volume; eGFR, estimated glomerular filtration rate; CHD, Coronary Heart Disease.

### Association Between the NCDRS and T2DM Risk

**FIGURE 1:**
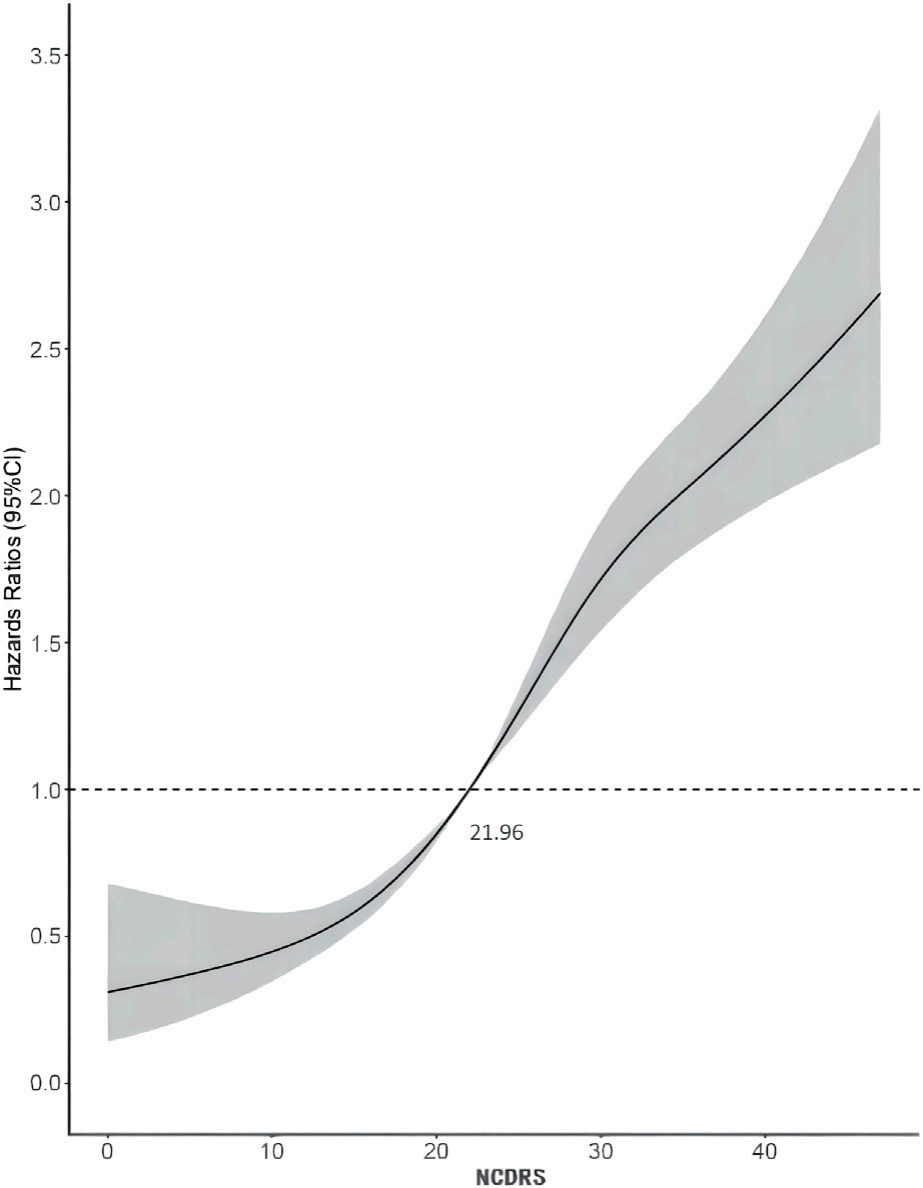
Dose-response relationship between the New Chinese Diabetes Risk Score and type 2 diabetes mellitus risk (Beijing, China. 2009–2016).

**FIGURE 2:**
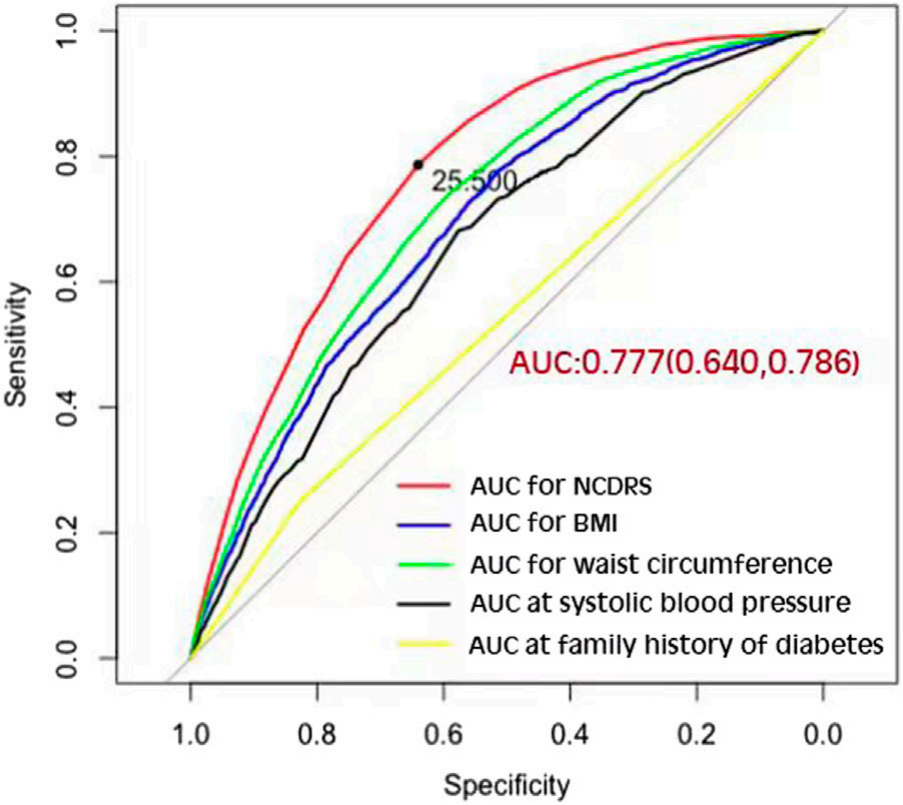
The area under the curve of the New Chinese Diabetes Risk Score compared with body mass index, waist circumference, systolic blood pressure, and family history of diabetes (Beijing, China. 2009–2016).

Of the 39,538 participants, 2,050 (74.10% men) developed T2DM during the follow-up duration of 141,481.43 person-years. As shown in Table 2, the incidence rates of T2DM according to the NCDRS quartiles were 0.146, 0.457, 1.394, and 3.268 per 100 person-years, respectively. The risk of T2DM was significantly increased with increasing NCDRS quartiles in all 3 models (*P* for trend <0.001). In Model 3, the highest HRs of T2DM risk were 1.76 (95% CI 1.25–2.47), 3.08 (95% CI 2.25–4.23), and 4.56 (95% CI 3.33–6.24) in the NCDRS of Q2, Q3, and Q4 (VS. Q1), respectively. Compared with the NCDRS <25 group, the HR of T2DM risk in the NCDRS ≥25 group was 2.12 (95% CI 1.88, 2.39). The HR for the risk of T2DM per SD increase in NCDRS was 1.60 (95% CI 1.51, 1.71). Furthermore, the restricted cubic spline (RCS) showed that the dose‒response association between the baseline NCDRS and T2DM risk was significantly nonlinear (*P*_nonlinearity_ <0.001; Figure 1). In fact, the dose‒response curve was shaped like an S. Additionally, the AUC of the NCDRS was 0.777 (95% CI 0.640–0.786), showing the best performance among the single indicators (BMI, waist circumference, systolic blood pressure, and family history of diabetes) of the NCDRS, and the cutoff point of the NCDRS was 25.50 (Figure 2).

**TABLE 2:** Multivariate models investigating the association between the New Chinese Diabetes Risk Score and risk of type 2 diabetes mellitus (Beijing, China. 2009–2016).

NCDRS	Person-year	No. of T2DM	Incidence (per 100 person-years)	HR (95% CI)		
				Model 1^a^	Model 2^b^	Model 3^c^
Q1	30,162.931	44	0.146	1(References)	1(References)	1(References)
Q2	31,705.361	145	0.457	3.06 (2.18, 4.29)	1.91 (1.36, 2.69)	1.76 (1.25, 2.47)
Q3	39,528.511	551	1.394	9.20 (6.77, 12.51)	3.55 (2.60, 4.85)	3.08 (2.25, 4.23)
Q4	40,084.631	1,310	3.268	21.44 (15.87, 28.95)	5.21 (3.83, 7.09)	4.56 (3.33, 6.24)
*P* for trend				<0.001	<0.001	<0.001
<25	79,116.353	756	0.469	1(References)	1(References)	1(References)
≥25	62,365.081	1,294	2.692	5.58 (4.99, 6.25)	2.25 (2.00, 2.54)	2.12 (1.88, 2.39)
Per SD increase				2.74 (2.59, 2.89)	1.63 (1.54, 1.73)	1.60 (1.51, 1.71)

^a^ Model 1: Unadjusted.

^b^ Model 2: Adjusted for Glu, smoking status, and alcohol consumption.

^c^ Model 3: Further adjusted for TG, TC, HDL, ALT, AST, eGFR, UA, heart rate, and white blood cell count (WBC) and all covariates in Model 2.

### Subgroup Analysis of the Association Between the NCDRS and T2DM Risk

We also performed subgroup analyses stratified by sex, hypertension (yes or no), and fatty liver disease (yes or no), to ascertain whether the association between the NCDRS and the risk of T2DM was stable in different groups, and these results confirmed that the association was consistent, as provided in Table 3. A similar trend (*P* for trend <0.001) by sex, hypertension, and fatty liver disease was also found in the association between the NCDRS and the risk of T2DM, the risk of T2DM was significantly increased with the increasing NCDRS.

**TABLE 3:** Subgroup analysis for the association between the New Chinese Diabetes Risk Score level and risk of type 2 diabetes mellitus (Beijing, China. 2009–2016).

NCDRS	Person-year	No. of T2DM	Incidence (per 100 person-years)	HR (95% CI)		
				Model 1^a^	Model 2^b^	Model 3^c^
Male						
Q1	7,531.031	11	0.146	1(References)	1(References)	1(References)
Q2	16,520.778	84	0.508	2.66 (1.74, 4.07)	1.75 (1.14, 2.67)	1.80 (1.17, 2.78)
Q3	27,039.147	374	1.383	9.24 (6.37, 13.40)	3.93 (2.69, 5.74)	4.03 (2.71, 5.98)
Q4	31,706.269	1,050	3.312	20.05 (13.95, 28.82)	5.44 (3.73, 7.95)	5.65 (3.76, 8.47)
*P* for trend				<0.001	<0.001	<0.001
<25	35,099.083	211	0.601	1(References)	1(References)	1(References)
≥25	47,698.142	1,308	2.742	6.67 (5.54, 8.03)	2.74 (2.25, 3.34)	2.66 (2.15, 3.28)
Per SD increase				3.00 (2.72, 3.30)	1.79 (1.61, 1.99)	1.80 (1.60, 2.03)
Female						
Q1	22,631.900	33	0.146	1(References)	1(References)	1(References)
Q2	15,184.583	61	0.402	3.42 (1.83, 6.42)	2.22 (1.18, 4.17)	2.04 (1.09, 3.84)
Q3	12,489.364	177	1.417	9.18 (5.04, 16.73)	3.71 (2.03, 6.77)	3.32 (1.81, 6.08)
Q4	8,378.361	260	3.103	21.84 (12.06, 39.57)	5.63 (3.09, 10.23)	5.23 (2.87, 9.55)
*P* for trend				<0.001	<0.001	<0.001
<25	44,017.269	160	0.363	1(References)	1(References)	1(References)
≥25	14,666.939	371	2.529	4.47 (3.86, 5.17)	2.01 (1.73, 2.34)	2.02 (1.73, 2.35)
Per SD increase				2.59 (2.41, 2.77)	1.59 (1.47, 1.71)	1.63 (1.51, 1.77)
Hypertension (no)						
Q1	30,056.931	44	0.146	1(References)	1(References)	1(References)
Q2	30,477.169	132	0.433	2.88 (2.04, 4.05)	1.72 (1.22, 2.43)	1.53 (1.08, 2.17)
Q3	32,080.808	409	1.275	8.34 (6.11, 11.38)	2.96 (2.15, 4.06)	2.46 (1.78, 3.41)
Q4	18,132.903	515	2.840	18.37 (13.49, 24.99)	4.21 (3.06, 5.8)	3.54 (2.55, 4.92)
*P* for trend				<0.001	<0.001	<0.001
<25	75,615.661	329	0.435	1(References)	1(References)	1(References)
≥25	35,132.150	771	2.195	4.87 (4.28, 5.55)	1.96 (1.71, 2.25)	1.82 (1.58, 2.09)
Per SD increase				3.04 (2.81, 3.28)	1.68 (1.55, 1.83)	1.62 (1.48, 1.78)
Hypertension (yes)						
Q1&Q2	1,334.192	13	0.974	1(References)	1(References)	1(References)
Q3	7,447.703	142	1.907	1.91 (1.08, 3.38)	1.56 (0.88, 2.75)	1.47 (0.83, 2.60)
Q4	21,951.728	795	3.622	3.62 (2.09, 6.27)	2.15 (1.24, 3.72)	2.07 (1.19, 3.59)
*P* for trend				<0.001	<0.001	<0.001
<25	3,500.692	42	1.200	1(References)	1(References)	1(References)
≥25	27,232.931	908	3.334	2.73 (2.00, 3.72)	1.83 (1.34, 2.5)	1.85 (1.36, 2.53)
Per SD increase				1.87 (1.68, 2.09)	1.37 (1.22, 1.53)	1.39 (1.24, 1.56)
Fatty liver disease (no)						
Q1	28,904.097	35	0.121	1(References)	1(References)	1(References)
Q2	25,368.922	81	0.319	2.57 (1.73, 3.81)	1.72 (1.15, 2.56)	1.68 (1.13, 2.51)
Q3	23,114.647	220	0.952	7.53 (5.27, 10.75)	3.27 (2.27, 4.7)	3.17 (2.18, 4.60)
Q4	16,570.086	365	2.203	17.26 (12.19, 24.43)	4.72 (3.29, 6.78)	4.49 (3.09, 6.52)
*P* for trend				<0.001	<0.001	<0.001
<25	65,121.833	186	0.286	1(References)	1(References)	1(References)
≥25	28,835.919	515	1.786	6.03 (5.10, 7.14)	2.53 (2.11, 3.02)	2.42 (2.01, 2.92)
Per SD increase				2.83 (2.59, 3.08)	1.70 (1.55, 1.87)	1.67 (1.51, 1.84)
Fatty liver disease (yes)						
Q1	1,258.833	9	0.715	1(References)	1(References)	1(References)
Q2	6,336.439	64	1.010	1.40 (0.69, 2.80)	1.07 (0.53, 2.16)	1.09 (0.54, 2.19)
Q3	16,413.864	331	2.017	2.74 (1.41, 5.32)	1.53 (0.79, 2.98)	1.52 (0.78, 2.95)
Q4	23,514.544	945	4.019	5.45 (2.82, 10.50)	2.13 (1.10, 4.12)	2.24 (1.16, 4.34)
*P* for trend				<0.001	<0.001	<0.001
<25	13,994.519	185	1.322	1(References)	1(References)	1(References)
≥25	33,529.161	1,164	3.472	2.58 (2.21, 3.02)	1.54 (1.32, 1.80)	1.61 (1.38, 1.89)
Per SD increase				2.04 (1.89, 2.21)	1.38 (1.27, 1.50)	1.45 (1.33, 1.58)

^a^ Model 1: Unadjusted.

^b^ Model 2: Adjusted for Glu, smoking status, and alcohol consumption.

^c^ Model 3: Further adjusted for TG, TC, HDL, ALT, AST, eGFR, UA, heart rate, and white blood cell count (WBC) and all covariates in Model 2.

### Sensitive Analysis

The increasing risk of T2DM with a higher NCDRS was also confirmed in the sensitivity analysis by excluding T2DM cases identified during the first 2 years of follow-up, and the adjusted HRs (95% CI) for T2DM risk were 1.73 (1.14–2.62), 2.70 (1.82–3.99), and 4.23 (2.87–6.23) in the second, third, and fourth (vs. the Q1) NCDRS quartiles, respectively, after adjusting for all covariates in Model 3 (Table 4).

**TABLE 4:** Sensitivity analysis of multivariate model investigating the association between the New Chinese Diabetes Risk Score and risk of type 2 diabetes mellitus (Beijing, China. 2009–2016).

NCDRS	Person-year	No. of T2DM	Incidence (per 100 person-years)	HR (95% CI)		
				Model 1^a^	Model 2^b^	Model 3^c^
Q1	26,413.683	29	0.110	1(References)	1(References)	1(References)
Q2	28,679.361	97	0.338	2.85 (1.88, 4.31)	1.91 (1.26, 2.90)	1.73 (1.14, 2.62)
Q3	35,923.603	321	0.894	7.20 (4.92, 10.53)	3.20 (2.18, 4.70)	2.70 (1.82, 3.99)
Q4	36,644.019	775	2.115	16.66 (11.50, 24.14)	5.00 (3.42, 7.30)	4.23 (2.87, 6.23)
*P* for trend				<0.001	<0.001	<0.001
<25	70,764.475	235	0.332	1(References)	1(References)	1(References)
≥25	56,896.192	987	1.735	4.78 (4.14, 5.51)	2.21 (1.90, 2.56)	2.05 (1.76, 2.39)
Per SD increase				2.57 (2.40, 2.76)	1.66 (1.54, 1.79)	1.62 (1.50, 1.76)

^a^ Model 1: Unadjusted.

^b^ Model 2: Adjusted for Glu, smoking status, and alcohol consumption.

^c^ Model 3: Further adjusted for TG, TC, HDL, ALT, AST, eGFR, UA, heart rate, and white blood cell count (WBC) and all covariates in Model 2.

*Sensitivity analysis was performed by excluding patients diagnosed with T2DM within the first 2 years of follow-up.

## Discussion

From the 7-year long large prospective longitudinal cohort study to investigate the association between the NCDRS and the risk of T2DM, we found that there was a significant association between the increased NCDRS and the increasing risk of T2DM, and a significant S-shaped nonlinear dose-response relationship was observed between the NCDRS and T2DM risk. Additionally, the AUC of 0.777 with the best cut off point of 25.50 supported the best performance of NCDRS in predicting the risk of T2DM among Chinese population. Based on a large nationwide diabetes cross-sectional study conducted in 12 provinces and autonomous regions as well as 2 municipalities in China, the NCDRS was developed under the estimated coefficient of candidate diabetic risk factors in a regression analysis of 41,809 participants (19). By computing 6 independent non-laboratory indicators (age, BMI, sex, waist circumference, SBP, and family history of diabetes), the total score of each participant was used to assess the risk of T2DM (19). The NCDRS ranging from 0–51 with an optimal cutoff point of 25 was reported to be a reliable screening tool for detecting T2DM in the Chinese population (19, 21, 23). Our findings of a positive significant association between the NCDRS and T2DM risk are consistent with previous studies (20, 21, 23).

When assessing the association between the NCDRS and the risk of T2DM, pre-diabetes is a non-negligible confounder that might impact the effect size. Previous studies showed that among adults diagnosed with prediabetes, approximately 70% progressed to T2DM within a decade (27). After excluding individuals who were diagnosed with T2DM in the first 2 years of follow-up from the sensitivity analysis, the association between the NCDRS and the risk of T2DM was slightly lower than the association in the original multivariable Cox proportional hazards models, which confirmed that prediabetes indeed exaggerated the relationship between the NCDRS and the risk of T2DM. Furthermore, a nonlinear association between the NCDRS and the risk of T2DM was found through a restricted cubic spline model, and in line with our Cox regression results, the risk of T2DM increased with an increasing NCDRS score. While the recommended cutoff point of the NCDRS was 25 (19), in our study, we found that the risk of T2DM sharply increased with NCDRS scores greater than 21.96. Considering this, future studies with larger sample sizes and longer follow-up periods need to be conducted to verify whether adults with an NCDRS score greater than 21.96 deserve attention in community T2DM prevention programs.

In the present prospective cohort study, the AUC of the NCDRS to predict the risk of T2DM was 0.777, which indicated it performed better than in the original population (19). The AUC in this study is larger than in other previous studies of the NCDRS for identifying T2DM (20, 21, 28), indicating that the NCDRS is a reliable tool for T2DM prediction in the Chinese population. Apart from this, we also found that the cutoff point in this population was 25.5, which is close to, but a half-point slightly higher than the recommended cutoff point from the original study (19). However, the cross-sectional studies conducted in Eastern and Southwest Chinese populations reported that the best cutoff points of the NCDRS for detecting T2DM were 27 and 28, respectively, which are much higher than those in our study (20, 23). These variations among different studies could be explained as follows: first, the optimal cutoff point might be a result of the different average ages for each population among these studies, and individuals with older ages have a higher cutoff point for T2DM detection (28). Second, differences among regions could be a critical factor in identifying T2DM, suggesting that studies of the NCDRS in different regions of China are required (28). In addition, the AUCs of the various components of the NCDRS are smaller than the AUC of the NCDRS for detecting T2DM. Thus, the NCDRS as a comprehensive risk score model is more effective for T2DM prediction than single indicators such as BMI, waist circumference, systolic blood pressure, and family history of diabetes.

The mechanisms of how obesity is associated with T2DM risk have been recognized. Obesity induces low-level inflammation in various tissues (e.g., adipose tissue, liver, pancreas islet, and brain), and obesity-related immune cells accumulate and engage in inflammatory polarization, contributing to obesity-linked metabolic dysfunctions, resulting in insulin resistance and type 2 diabetes mellitus (29, 30). Therefore, obesity is the most important predictor of T2DM in NCDRS. Given that BMI and waist circumference (WC) are basic anthropometric indicators of obesity and abdominal obesity (31), participants with higher BMI or WC had an increased predisposition to T2DM (32). Likewise, some unhealthy lifestyle behaviors, such as more frequent smoking or high alcohol consumption are more common in Chinese men, which possibly exerts sex-dimorphic effects on glucose metabolism leading to gender differences in the risk of T2DM (33, 34). Unsurprisingly, aging is another typical factor contributing to T2DM, especially after age 40 (35), and a proinflammatory state as an inevitable age-related condition inducing insulin resistance, might shed light on the higher risk of T2DM as age increases (32, 36). Furthermore, hypertension and T2DM are common comorbidities (37). Several longitudinal studies found that participants with hypertension or prehypertension were at high risk of developing diabetes mellitus (38), since hypertension patients always exhibit insulin resistance, which increases the risk of T2DM(37), and SBP control is associated with a lower risk of diabetes mellitus (39). On the other hand, the association between SBP and T2DM complications of renal dysfunction (40) and vision problems means SBP is a significant factor in T2DM prediction. Similarly, commonly identified genetic variants in successive genome-wide association studies (GWASs) have helped to explain the increased susceptibility of participants with a family member with T2DM (41). Thus, the NCDRS—a comprehensive tool reflecting T2DM risk—might be useful for T2DM screening in the Chinese population.

In our study, we also conducted stratified analyses by sex, hypertension, and fatty liver disease at baseline. Fatty liver disease is a confounder of the association between the NCDRS and the risk of T2DM. A five-year cohort study conducted in Northern China reported that fatty liver disease was an indicator in T2DM prediction (42). Likewise, the causal relationship between fatty liver disease and T2DM was also concluded in a meta-analysis (43). However, we found a stronger association between the NCDRS and T2DM in non-fatty liver disease participants than in the fatty liver disease population, which was contradictory to the previous study. An explanation for this could be that aiming to decrease liver fat contributes to preventing T2DM, and patients who had fatty liver disease might take more lipid-regulatory drugs to decrease liver fat, potentially leading to the decrease of T2DM risk (44). Further research with larger samples of the subgroup analyses are needed to validate the association between the NCDRS and the risk of T2DM in participants with or without fatty liver disease.

Several limitations to the current study should be considered. First, the data for our study were obtained from a local rehabilitation hospital in Beijing, whose patients are mostly employees of local governmental organizations, lacking representation of the general population. Therefore, further evidence from different types of T2DM patients is needed to confirm our findings, and additional studies are needed to confirm whether the NCDRS is useful for T2DM screening for other populations of different ethnicities. Second, reporting bias could not be overlooked when conducting face to face interviews for participants who had difficulty completing the survey. Moreover, other unmeasured related confounders, such as the frequency of physical activity, and dietary intake habits, should be taken into consideration to estimate the association between the NCDRS and the risk of T2DM.

Despite the limitations mentioned above, this study also provides some practical strengths for the NCDRS in predicting T2DM risk. First, we add new evidence that the association between the NCDRS and T2DM risk is nonlinear and shaped like an S. Second, as the NCDRS includes only easily measured factors which do not require laboratory work, and has great performance, it might be suitable for the population of T2DM screening under limited health resource conditions in most of China. Additionally, considering that our study was based on annual check-up data mostly from employees of local governmental organizations, the NCDRS could be a simple, practical model in T2DM screening for this certain occupational population to identify their risk of T2DM by using electronic health records by general practitioners, with a reference of the optimized cutoff derived from this study, to help with further T2DM intervention programs.

### Conclusion

In conclusion, the NCDRS is significantly associated with the risk of T2DM, and is a valid predictive index for screening the population for undiagnosed T2DM in China. It could be suitable to implement in T2DM screening programs across a wide range of Chinese populations.

## Abbreviations

ALT, alanine aminotransferase; AST, aspartate aminotransferase; BMI, body mass index; CIs, Confidential Intervals; Cr, creatinine; DBP, diastolic blood pressure; eGFR, estimated glomerular filtration rate; EOS, eosinophil; EOSP, eosinophil percentage; ESR, erythrocyte sedimentation rate; FLD, fatty liver disease; FPG, fasting plasma glucose; GWASs, genome-wide association studies; HC, hip circumference; HDL-C, high-density lipoprotein cholesterol; HRs, Hazard Ratios; IQR, interquartile range; LDL-C, low-density lipoprotein cholesterol; MCV, mean corpuscular volume; NCDRS, The New Chinese Diabetes Risk Score; RCS, The restricted cubic splines; SBP, systolic blood pressure; SD, standard deviation; T2DM, Type 2 Diabetes Mellitus; TC, total cholesterol; TG, triglycerides; UA, serum uric acid; WBC, white blood cell count; WC, Waist circumference.
